# A Remarkable Fuel

**Published:** 1874-11

**Authors:** 


### A REMARKABLE FUEL.

A fuel is now offered for sale in the various cities along the Atlantic seaboard, which, it is claimed, possesses some qualities of excellence to commend it to all. It is a natural product of Virginia, and yet it is neither wood, nor lignite, nor anthracite, nor bituminous coal. It is quite anomalous in its character, and has no exact counterpart among known minerals. Its principal constituent being fixed carbon, it has received the appropriate name of "Carbonite."

In appearance this fuel is more like coke than anything else. But this likeness depends solely upon its color and its semi-metallic lustre. It is much heavier and denser than coke, and lacks the porosity of that substance. It leaves far less ashes after combustion; in fact, the ash remaining is so small, compared with that resulting from the combustion of all other fuel, as to call forth the remark, "it consumes its own ashes." Of course this statement cannot be strictly true, yet it is approximately correct. When carefully tested, one hundred pounds of the "Carbonite" is found to leave an ash-residuum of only two pounds and four ounces. Adding to this the fourteen and a quarter pounds of volatile combustible matter, the nearly eighty-two pounds of carbon—the equivalent of charcoal—and a trace of water and sulphur, the latter so small as to be detected only with difficulty—and we have the elements of this singular fuel.

The field supplying it is situated on the north side of the James River, about twelve miles from the city of Richmond. In that locality the article has been known and used for the last fifty years. It has long been looked upon as a very desirable heat-producer for manufacturing, not less than for domestic purposes. The heat which is obtained from its combustion is most intense, the experiments of Dr. Wallace, of Glasgow, proving it to possess greater heating power than the best anthracite.

We have endeavored to investigate its merits from a sanitary point of view, and we feel that we can earnestly recommend it on that score. We are not prepared to say that it is better than good, sound hickory wood, burnt in an open fire-place, but we believe it to be the next thing to it. In fact, it appears to possess one advantage over *all* other fuels. We allude to the fact that the ash resulting from its combustion is so heavy that it cannot be readily lifted into the air and scattered about the room; it is, in fact, about as heavy as sea-sand. This is of course a great advantage to those whose lungs are sensitive and liable to irritation from the inhalation of dust.

But its chief advantage rests in the fact that in burning it gives off no pungent and deleterious gases. An important product of combustion in the fuels in common use is carbonic-acid gas, which is a virulent poison when inhaled. It is this gas which destroys life in the fumes of burning charcoal. It is evolved, together with the scarcely less deadly sulphurous fumes, from burning anthracite. Next to alcohol, which is the great destroyer, this subtle gas is chargeable with the destruction of more human lives than all the other poisons known to science. If it is thrown off by the combustion of the "carbonite," the fact is not apparent to the senses.

This fuel has only been known in New York for a year or two, but its excellent qualities have secured for it a large sale. It is found to be adapted alike to furnaces and stoves, while the most favorable results are secured by its use in open grates. For cooking stoves and ranges it appears to give unlimited satisfaction.

The best test of its character is found in the reputation it has won wherever it has been used. Mr. Waite, one of the proprietors of the Brevoort House, informs us that an experiment tried last winter convinced him that this fuel was the most desirable to be had; he has accordingly provided it for all the open fires in his hotel. He declares that the saving in carpets, furniture and lace curtains, which is secured from the freedom from smoke and dust, added to the comfort of the guest in the matter of the absence of noisome and unwholesome gases and offensive odors, combine to render this the choice fuel of the period.

To sick people this substance would seem to be a peculiar boon. We have conversed with Mr. J. Silsby, of No. 49 New street, a prominent citizen of New York, who recites some remarkable facts concerning the "Carbonite." Last winter his daughter was suffering hemorrhage from the lungs, with the attendant difficulty of breathing. The furnace heat proved to be insupportable to her, owing to the gases which attended the combustion of anthracite. The new fuel was employed in the furnace, and the air was found to be pleasant and acceptable to the diseased lungs.

It would seem, then, that this fuel is destined to be very largely consumed by the best classes. Its freedom from soot and smoke render it more desirable than the cannel coal, while its cost is scarcely more than half that of the imported article, and its enduring qualities are superior even to those of anthracite. On the whole we consider this "Carbonite" the best fuel now attainable, and we believe its general use will save our readers from the payment of many a doctor's bill.

